# Otoliths suggest lifespans more than 30 years for free‐living bowfin *Amia calva*: Implications for fisheries management in the bowfishing era

**DOI:** 10.1111/jfb.15201

**Published:** 2022-09-08

**Authors:** Alec R. Lackmann, Ewelina S. Bielak‐Lackmann, Malcolm G. Butler, Mark E. Clark

**Affiliations:** ^1^ Department of Biology University of Minnesota Duluth Duluth Minnesota USA; ^2^ Department of Biological Sciences North Dakota State University Fargo North Dakota USA

## Abstract

The bowfin *Amia calva* is an amiid (Amiiformes) relict native to North America. It is the last surviving member of the Halecomorphi, a group of fishes that evolved more than 250 million years ago. Despite the phylogenetic significance of the amiids in vertebrate evolution, little has been published about their age and growth. Recreational bowfin harvest is currently unregulated throughout most of the USA, yet new recreational fisheries are emerging. As such, bowfin are increasingly harvested by sport bowfishing without limit, in addition to their growing commercial harvest for caviar. From 2017 to 2021 we studied a total of 81 bowfin from 11 populations across the east–west gradient of Minnesota within a narrow latitudinal margin (<50 km) of the 46th parallel north. We compared the allometry and translucence of bowfin asteriscus, lapillus and sagittal otoliths and found the lapillus otoliths provide consistent readability for age estimation despite being the smallest of the set. Size‐at‐age data derived from otoliths indicated that bowfin are sexually dimorphic in asymptotic length and may live up to 33 years, which is 15 years longer than previously estimated in wild populations, but comparable to what has been reported in captivity. Overall, 28% of the otolith‐aged fish were estimated as older than the previously reported maximum age for wild bowfin populations. Our findings suggest that the bowfin life history may exhibit slower growth, greater longevity, and more variable recruitment than previously recognized, which sets the stage for more otolith‐derived population demographics across their range and age validation study. Our results have direct implications for conservation of bowfin, especially amidst the increasing rates of exploitation during the bowfishing era.

1

**STATEMENT OF SIGNIFICANCE**

Bowfin otoliths have been abandoned in previous age studies. We present the first thorough analysis of all Bowfin otoliths and find that their lapilli and asterisci are especially useful for age estimation despite being smaller than the sagittae. Using otolith‐derived data we find that Bowfin may live 33 years, which is ~2–3 times longer than previously estimated for wild populations, but comparable to known‐age Bowfin lifespans in captivity. Our findings are especially relevant because Bowfin are becoming sportfish *via* bowfishing while also subject to a growing caviar fishery.

## 2 INTRODUCTION

Bowfin *Amia calva* is a fish species native to North America that is the only extant member of the order Amiiformes (Burr & Bennett, 2014). The bowfin is a top predator that tends to inhabit clear waters in vegetated bays (Carlander, 1969) that likely plays a crucial role in maintaining balance of its prey (Scarnecchia, 1992). The bowfin is capable of enduring variation in the quality of its habitat in part by obtaining oxygen from air ingested into the swim bladder at water temperatures above 10°C, with reports of individuals surviving weeks in saturated sediment lacking standing water (Becker, 1983). Bowfin provide parental care to their offspring, with males building a nest and guarding their brood for up to 2 months (Becker, 1983). Understanding the natural history of bowfin has considerable scientific value because their unique lineage holds clues to the evolution of vertebrates (Thompson *et al*., 2021). The bowfin is still known from all the regions where it was originally documented (Burr & Bennett, 2014).

Human exploitation of bowfin has increased in the 21st century. Over the past two decades there has been increased demand for bowfin roe (Davis, 2006; Koch *et al*., 2009b; Porter *et al*., 2014; Sinopoli & Stewart, 2021), increased participation in recreational angling for bowfin (Koch *et al*., 2009b; Porter *et al*., 2014), and increased harvest of bowfin through modern bowfishing (Lackmann *et al*., 2022, see Figure S1b; Scarnecchia & Schooley, 2020; Scarnecchia *et al*., 2021). Although the rate of exploitation appears to be increasing, much of the ecology of bowfin remains unknown. Indeed, Scarnecchia (1992) argued that the ecological function of bowfin has to be reassessed because this fish has long been subject to systemic neglect stemming from the ‘rough fish’ label.

Few bowfin populations have been investigated to quantify population demographics (Porter *et al*., 2014). Estimates of age at sexual maturity, mortality rate, lifespan, recruitment and growth require accurate age data. Age validation is always a goal, and in its absence age estimates should be reported with caution (Beamish & McFarlane, 1983). Although bowfin have been studied for age and growth determination, no age estimates by any method have been validated for this species. Despite several attempts (Cartier & Magnin, 1967; Davis, 2006; Koch *et al*., 2009a), otoliths have also not been used successfully for age estimation in this species. Thus, previous characterizations of bowfin demography have relied on age estimation using scales, vertebrae, opercula, gular plates or fin rays (Cartier & Magnin, 1967; Cooper & Schafer, 1954; Daniels, 1993; Davis, 2006; Hausmann, 1998; Holland, 1964; Koch *et al*., 2009a; Mundahl *et al*., 1998; Porter *et al*., 2014; Sanderson‐Kilchenstein, 2015; Schiavone Jr., 1982; Table 1). Based on these studies, it is presumed that bowfin live approximately a decade under natural conditions. However, hard body parts other than otoliths have been shown to underestimate ages in other species (Beamish & McFarlane, 1983; Campana, 2001; Casselman, 1990; Lackmann, Kettelhut, *et al*., 2021; Radford *et al*., 2021). Interestingly, there are at least three cases of captive bowfin living longer than any bowfin ever recorded in the wild: a 20‐year‐old (Flower, 1925), a 24‐year‐old (Flower, 1935) and a specimen in the New York Aquarium that lived for 30 years (Breder, 1936; reviewed by Randall & Delbeek, 2009). These captive bowfin of known age contradict the life expectancy estimated for wild populations using non‐otolith structures across their range.

**TABLE 1:** Review of age and growth studies on bowfin *Amia calva* compared to the present study

Reference	Structures attempted	Structures used	Age range (years)	TL range (cm)	*n*	Region
Cooper and Schafer (1954)	Scales	Scales	3–7	~47–65	39	Michigan
Holland (1964)	Scales, vertebrae, opercula, branchiostegal rays, fin rays, gular plates	Gular plates	1–10	NA	178	Missouri
Cartier and Magnin (1967)	Scales, gular plates, otoliths	Scales	1–9	22.1–71.8	80	Quebec
Schiavone (1982)	Scales	Scales	1–8	~33–73	20	NY
Daniels (1993)	Gular plates	Gular plates	1–3	26.2–49.5	37	WV
Mundahl *et al*. (1998)	Scales	Scales	7–18	51.5–77	48	MN
Hausmann (1998)	Gular plates	Gular plates	1–6	51.6–73	38	Illinois
Davis (2006)	Gular plates, otoliths	Gular plates	1–10	31.6–73.6	288	LA
Koch *et al*. (2009)	Fin rays, gular plates, scales, otoliths, branchiostegal rays	Fin rays	1–13	39.2–80.7	255	Iowa
Porter *et al*. (2014)	Fin rays	Fin rays	0–5	23.3–68.3	82	Georgia
Sanderson‐Kilchenstein (2015)	Fin rays	Fin rays	1–8	28.9–79.1	51	NY
This study	Otoliths	Otoliths	2–33	40.1–72.7	81	MN

*Note*: The information from Holland (1964) was gleaned from the review by Sanderson‐Kilchenstein (2015) and Koch *et al*. (2009). WV, West Virgina; NY, New York; MN, Minnesota; LA, Louisiana.

In this study we investigated the utility of all three pairs of bowfin otoliths for age estimation, characterized bowfin body growth and analysed recruitment. We quantified age demographics based on presumed annuli in thin‐sectioned otoliths and differences in otolith allometry among the three otolith types to inform otolith type usage for age estimation. Based on our age estimates, we modelled the first otolith‐derived growth characteristics of bowfin and examined variation in recruitment using catch curve analysis, offering a fresh perspective on their biology.

## 3 MATERIALS AND METHODS

### 3.1 Sample collection

**FIGURE 1:**
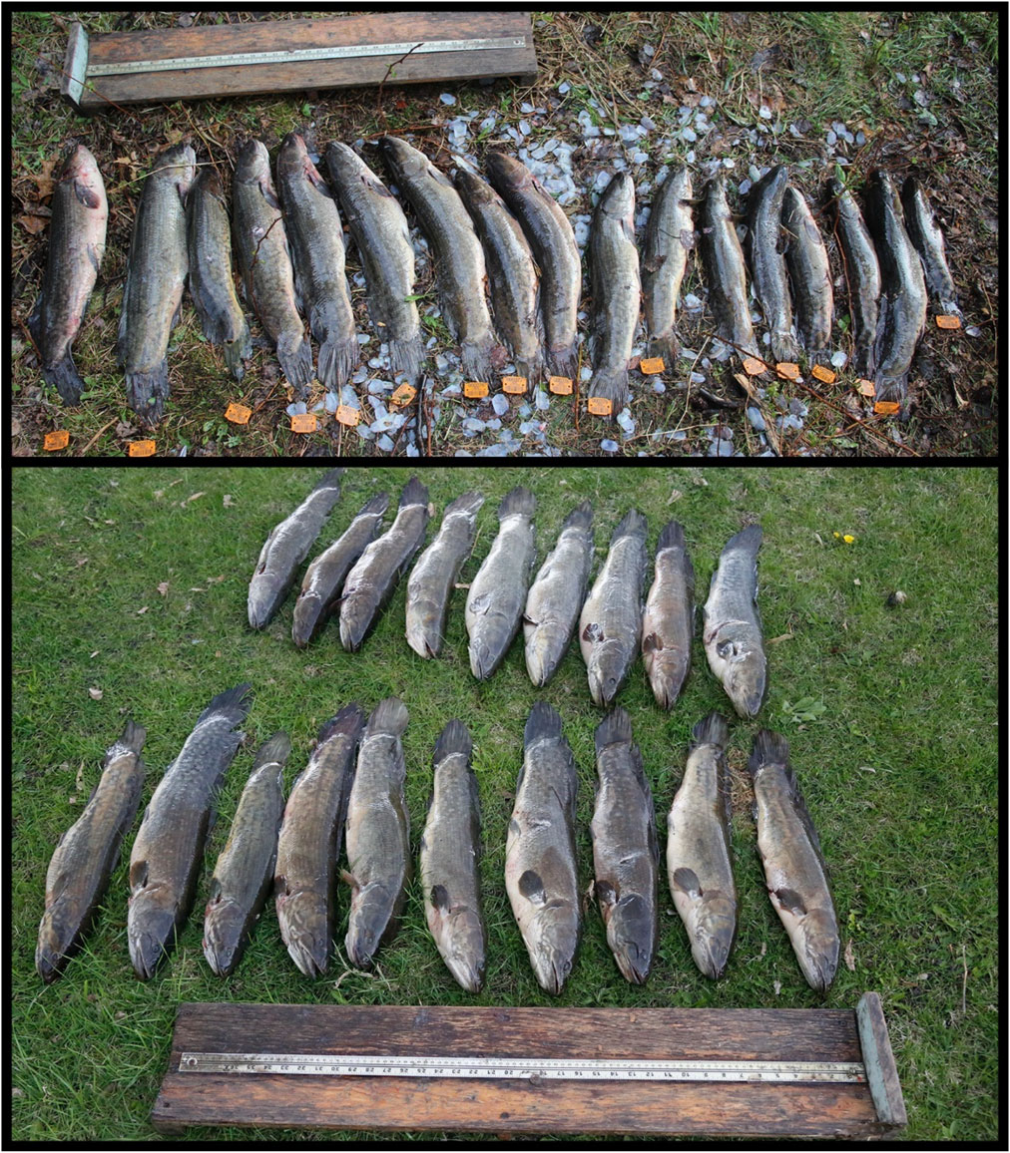
Representative harvest of bowfin (top and bottom panel) collected *via* bowfishers from two nights of bowfishing in spring 2021 in Minnesota. These fish (*n* = 36) ranged from 40.1 to 70.3 cm total length, and 558–2667 g in mass. Fish board in each panel = 1 m

From August 2017 to October 2021 we collected 81 bowfin from night bowfishing discards (Figure 1) in Minnesota. For the bowfishing groups with which we collaborated, bigmouth buffalo (*Ictiobus cyprinellus*), common carp (*Cyprinus carpio*) and white sucker (*Catostomus commersonii*) were the preferred targets, with bowfin being opportunistic targets for the groups. Nonetheless, opportunistic bowfishing was effective at harvesting bowfin (Figure 1).

We collected bowfin from 11 sites across east–west Minnesota, but within a narrow latitudinal gradient (<50 km). From north‐western MN, lakes sampled included North Lida in 2017 (46°35′01.3″N, 95°58′32.2″W; *n* = 1), West Lost in 2018 (46°23′00.1″N, 95°52′25.0″W; *n* = 1) and Little Pelican (46°42′38.6″N, 95°57′25.4″W; *n* = 5) and Rush (46°36′41.1″N, 95°59′25.3″W; *n* = 2) in 2020–2021. From the northcentral region of the state, sites included the Mississippi River near Riverton (46°26′49.9″N, 94°05′50.5″W; *n* = 11) in 2018–2021, Lower Cullen Lake (46°32′19.6″N, 94°17′30.1”W; *n* = 10) in 2019–2020 and Clark Lake (46°30′36.5″N, 94°15′49.8”W; *n* = 1) in 2021. Finally, from north‐eastern MN, lakes sampled included Lower Island (46°39′57.5″N, 92°52′31.8”W; *n* = 3), Tamarack (46°40′01.3″N, 92°58′55.4″W; *n* = 16), Big Sandy (46°46′44.5″N, 93°19′10.1″W; *n* = 30) and Cedar (46°31′05.9″N, 93°47′08.9”W; *n* = 1) in 2021.

### 3.2 Body dissections

We measured the size of individual fish, and dissected to extract otoliths and determine sex *via* gonadal examination. We quantified fish size by wet mass (±1 g) and total length (±1 mm) either immediately after the fish was landed or within a few hours after the fish had expired. For those specimens in which wet mass was quantified a few hours of harvest, fish were again immersed in water for approximately a minute prior to obtaining mass. After extraction we placed otoliths immediately in microvials pre‐filled with distilled water. Because we were initially focused on only extracting the largest otoliths from bowfin, for eight individuals we extracted only sagittal otoliths, but for the remaining 73 fish we extracted sagittal, asteriscus and lapillus otoliths.

### 3.3 Otolith analysis

**FIGURE 2:**
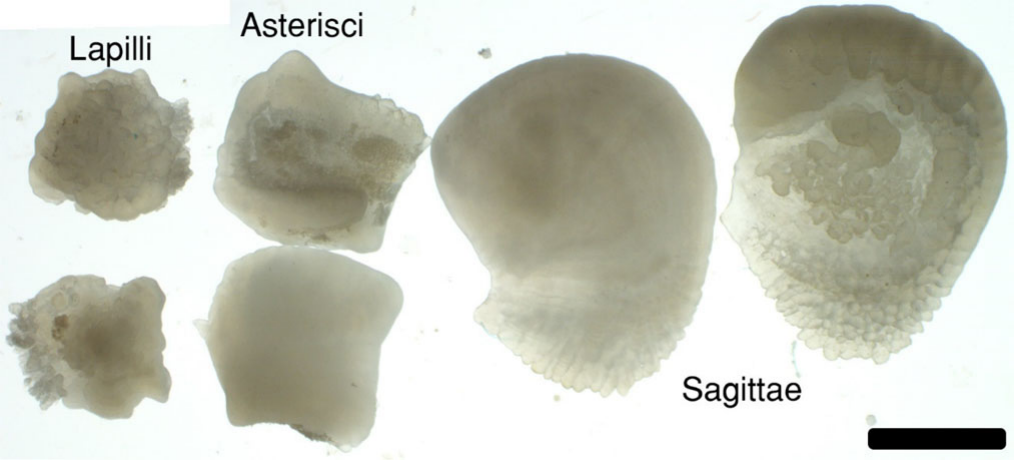
An otolith set from an individual bowfin *Amia calva*. Although lapillus otoliths are the smallest pair in this species, they were most useful in estimating age. Scale bar = 3 mm

In the laboratory we processed extracted otoliths to obtain photographs of their whole structure and measurements of mass. We removed residual cranial tissue and other non‐otolith material under a dissecting microscope. We photographed whole otoliths in water under a dissecting microscope at 50×, using transmitted light in light‐field mode (Figure 2). Inspection and photography under a dissecting microscope helped determine the location of the core and primary growth axis of each otolith type prior to sectioning. We air‐dried otoliths and measured sagittal, asteriscus and lapillus otolith mass on a microbalance (±0.1 mg). A few otoliths were sectioned without obtaining an otolith mass because we had yet to adopt otolith weighing as standard protocol during pioneering stages of the study. In individuals for which both otoliths of a pair were measured for mass, we used the greatest mass of the two in subsequent analyses. We used this protocol because we assume minor amounts of otolith material could sometimes be lost during extraction and handling.

We thin sectioned processed otoliths for age analysis. We embedded otoliths in either ACE® quick‐setting or Buehler epoxy, then sectioned them using twin diamond‐embedded blades on a Buehler IsoMet™ 1000 low‐speed saw to produce 300 μm sections. We sectioned otoliths through the core and along the primary growth axis. We mounted sections on a glass slide, immersed them in mineral oil and photographed them at 75× on a compound microscope. Across all specimens (*n* = 81), we produced and aggregate total of 101 thin sections.

We analysed images of thin sections to estimate individual age. Presumed annuli were digitally marked on images by two independent readers following the age‐reading protocol in Lackmann *et al*. (2019). Each reader was experienced, having processed and age scored thousands of otolith thin sections across several dozen North American freshwater fish species, with considerable experience at age validation (Lackmann *et al*., 2019, 2021b). High‐resolution composite images of the entire thin section were age scored (each presumed annulus marked) along the primary growth axis of each thin‐sectioned otolith. If a thin section was deemed unreadable by the primary reader, then another otolith from that specimen was thin sectioned until a readable section was obtained. We assigned year classes to fish based on collection date and ages derived from the total presumed annuli marked on the thin‐sectioned otolith images. For each image, we determined whether the marginal increment on the outer edge of the otolith thin section should be counted as a presumed annulus or not by considering the date of capture relative to probable hatch date, that is, rounding the estimated age to the nearest year while presuming a probable hatch date of mid‐May because bowfin spawn once annually during spring at this latitude (Carlander, 1969; Cartier & Magnin, 1967).

### 3.4 Statistical analysis

We used the von Bertalanffy growth function (von Bertalanffy, 1938) to model length at age such that $\mathrm{TL}=L_{\infty }∙\left(1-e^{-k∙\left(\mathrm{age}-t_{0}\right)}\right)$, where TL is total length (mm), age is in years, *L*_*∞*_ is asymptotic total length (mm), parameter *k* is the instantaneous rate of increase (mm/mm/d) (Schnute & Fournier, 1980) and parameter *t*_0_ is age (years) at length 0. We also estimated $\omega =L_{\infty }∙k$, where *ω* is the early‐life growth rate, also known as the growth rate near *t*_0_ (Gallucci & Quinn, 1979). We developed four models for total length based on combinations of parameters *L*_*∞*_ and *k* that varied by sex, while *t*_0_ was fixed (at 0). We did not estimate *t*_0_ because the lack of individuals younger than 2 years of age would result in unrealistic (highly negative) *t*_0_ values. We used information‐theoretic methods (Burnham & Anderson, 2002) to determine the highest ranked models for size at age based on the relative Akaike's Information Criterion corrected for small sample sizes (ΔAICc) when comparing multiple models in the von Bertalanffy model suite (Akaike, 1973). To characterize otolith allometry, we used Analysis of Covariance (ANCOVA) to quantify the effects of (natural log‐transformed) body mass (kg), sex and their interaction on (natural log‐transformed) otolith mass (mg). Similarly, we used ANCOVA to quantify the effects of (natural log‐transformed) age (years), sex and their interaction on (natural log‐transformed) otolith mass (mg). We used JMP 16 Pro Statistical Discovery™ for statistical analysis and graphical output.

For recruitment analysis we used catch curves (Maceina, 2004) and the recruitment coefficient of determination (RCD; Isermann *et al*., 2002). We analysed only 2021 collected fish to standardize the age distribution, and we excluded year classes 2017–2021 or 2013–2021, depending on the analysis. We used two different exclusionary ranges based on alternative assumptions of the age at which bowfin recruit to the bowfishery (age of 5 years versus age of 9 years). RCD values range from 0 to 1 because this metric is the *R* ^2^ of a catch curve, with higher values indicating more stable recruitment (Isermann *et al*., 2002). We pooled bowfin across sites for both recruitment and growth analyses because this allowed for a large enough sample to be analysed for any general trends. We concluded this was warranted since this is the first such otolith‐derived data for bowfin, and because all bowfin were collected within a narrow latitudinal gradient (<50 km). Furthermore, 93% of the 2021 collected fish were from hydrologically connected waters along the Mississippi River, whereas the other 7% came from adjacent waters within the Pelican River watershed (Hudson Bay drainage). Nonetheless, the results of these growth and recruitment analyses should be interpreted with caution because they are potentially limited in scope.

## 4 RESULTS

**FIGURE 3:**
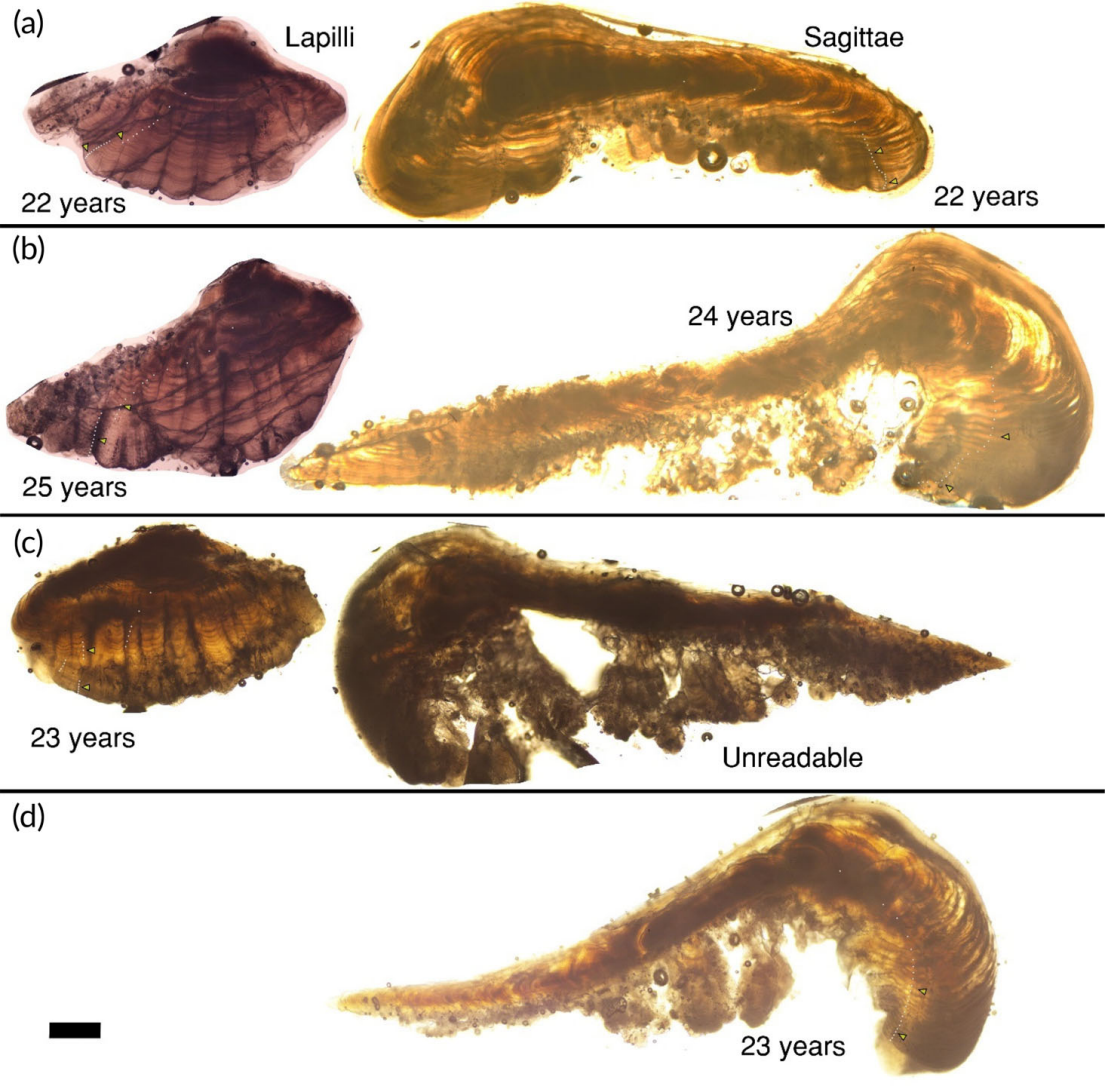
A comparison of lapillus versus sagittal otolith thin sections from bowfin *Amia calva*. (a–d) Images of thin‐sectioned otoliths from four individuals, three of which (a–c) had both otolith types sectioned. (a) Images from both sections are comparable in quality and yielded identical age estimates. (b) Presumed annuli in the image of the lapillus thin section are clearly defined, unlike presumed annuli in the image of the sagittal otolith section for the most recent years of growth, and the images yielded different age estimates. (c) The sagittal otolith thin section image was unreadable for this individual whereas the lapillus thin section image revealed consistent presumed annuli for an age estimate. (d) Although 40% (eight of the 20 sagittal otoliths sectioned) of sectioned sagittae were unreadable, some yielded clearly defined presumed annuli like this estimated 23‐year‐old specimen and are therefore potentially useful for age estimation if the lapillus otolith is damaged or lost during extraction. White dots denote presumed annuli; triangles mark decades. Scale bar = 600 μm for all

**FIGURE 4:**
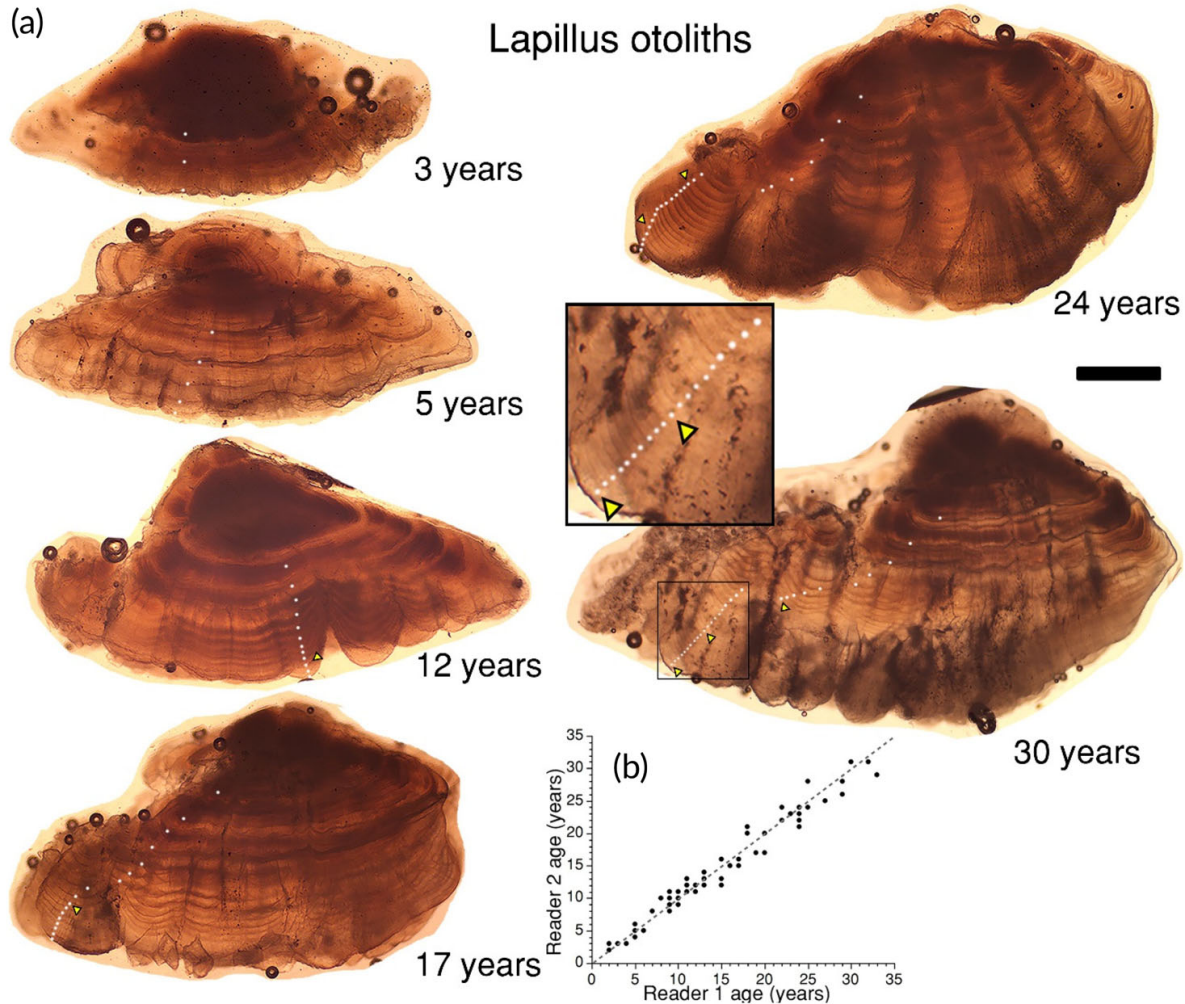
(a) Thin‐sectioned lapillus otoliths of Bowfin *Amia calva*. Examples range from 3 to 30 years. Dots mark each presumed annulus; triangles mark each decade. Note the inset for the estimated 30‐year‐old individual revealing the last 20 years of this fish's ontogeny. Scale bar = 600 μm (does not apply to inset or to the age bias plot). Note the well‐defined presumed annuli. (b) Age bias plot of the secondary *vs*. the primary reader for the lapillus otolith (*n* = 71) with the 1:1 line also shown for reference (dotted grey line). The Pearson correlation coefficient of Reader 2 *vs*. Reader 1 was an *r* = 0.98

**FIGURE 5:**
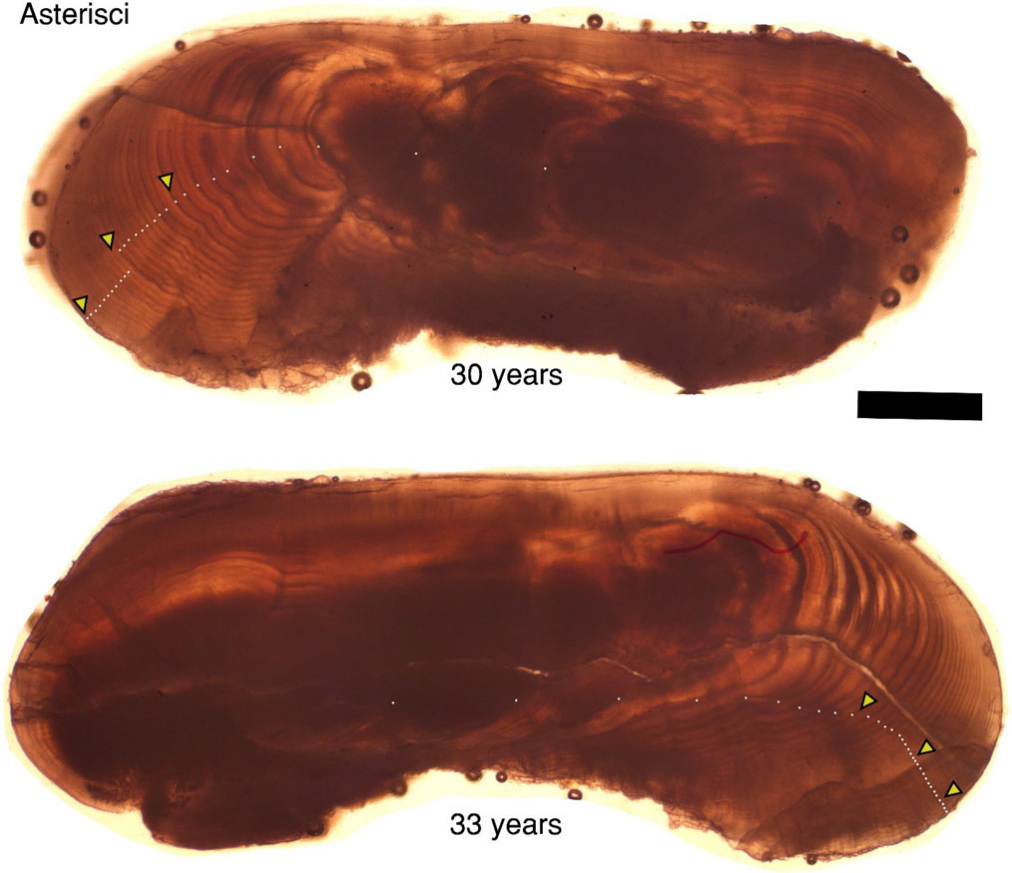
Thin‐sectioned asteriscus otoliths of Bowfin *Amia calva*. Examples of an estimated 30 and 33‐year‐old individual. The 30‐year‐old from Figure 4 (lapillus) is also the 30‐year‐old specimen in this figure (asteriscus). Dots mark each presumed annulus; triangles mark each decade. Scale bar = 600 μm. Note the well‐defined presumed annuli

We estimated the ages of 81 bowfin in this study from presumed annulus counts of the otolith thin sections, with ages ranging from 2 to 33 years old (*e.g*., Figures 3, 4, 5). The size of these individuals ranged from 40.1 to 72.7 cm TL and 558–3329 g in mass, with 56 females and 25 males. Across all three otolith types, between‐reader aging precision had a coefficient of variation (CV; Campana *et al*., 1995) of 6.3%. We sectioned 20 sagittae for which eight sections were unreadable. The CV for the readable 12 sagittae was 7.0% and these fish ranged from 5 to 23 years in age (*e.g*., Figure 3). Because 40% of the sectioned sagittae were unreadable, we examined the lapilli for age determination (*e.g*., Figure 4a). We sectioned 71 lapilli for which 100% were readable ranging from 2 to 33 years old with a CV of 6.2%. We also examined asterisci for age determination by sectioning a subsample (*n* = 10) and found 90% were readable ranging from 9 to 33 years old with CV of 6.1% (*e.g*., Figure 5). In cases where multiple otolith types produced readable sections from a given individual, the age scores were consistent across otolith types (*e.g*., Figure 3, 4, 5). For the lapillus otolith, the otolith type that was most used because it most consistently produced readable sections, an average percent error (APE) of 4.4% was calculated between reader age scores, while the overall agreement of age scores between the secondary and primary reader was a Pearson correlation coefficient = 0.98 (Figure 4a). More specifically, the same age score (exact agreement) was assigned to 30% of all lapillus otolith sections, 76% were in agreement to within 1 year (or fewer), while 90% were in agreement to within 2 years (or fewer), while the remaining 10% differed by 3 (*n* = 6) or 4 (*n* = 1) in absolute age score difference. The APE for the lesser‐used sagittal and asteriscus otoliths was 4.9% and 4.3%, respectively.

**FIGURE 6:**
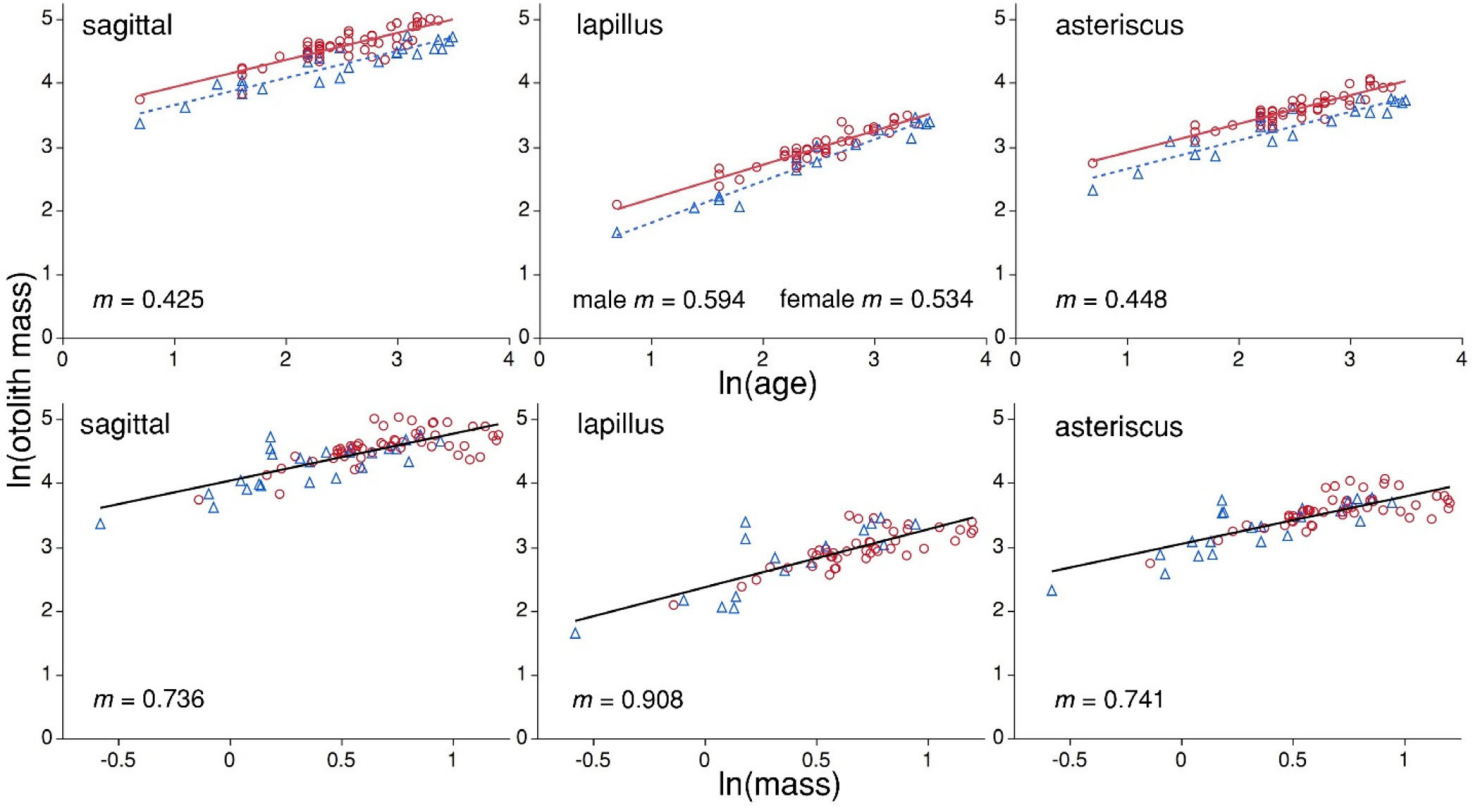
Allometry of bowfin *Amia calva* otoliths as a function of age (top panels) and body mass (bottom panels) of the final models (see results). Analysis of covariance of natural log‐transformed otolith mass (mg) versus natural log‐transformed age (years) and natural log‐transformed mass (kg) reveals otolith growth is proportionately greatest (as indicated by values of the slope) in the lapillus. Coefficient *m* = slope; circles and solid red line = females; triangles and dotted line = males; solid black line indicates no significant sex effect. See Table 2 for statistics

Analysis of covariance (ANCOVA) of otolith allometry indicated that the lapillus otolith grows proportionately more than the other otolith types (sagittal or asteriscus) in bowfin. We found that mass of the lapillus otolith increases at a greater rate than mass of the sagittal or asteriscus as bowfin increase in age or as they increase in total body size (Figure 6 and Table 2). We also found that estimated age explained more variation in otolith mass (83%–92% depending on otolith type) than did total body size (60%–62% depending on otolith type) and that it was mass of the lapillus otolith that explained the most variation in age (92%) compared to the other otolith types (Table 2).

**TABLE 2:** ANCOVA statistics for the natural log‐transformed otolith mass *vs.* natural log‐transformed age and body mass (BM) analysis (in conjunction with Figure 6)

Model	*n*	*I*	*m*	SexF	Sex*AgeF	*F*	*P*	*R* ^2^
Sagittal *vs*. age	80	3.4 (0.06)	0.426 (0.025)	0.14 (0.02)	NS	125	<0.001	0.83
Sagittal *vs*. age p.h.	80	3.4 (0.06)	0.425 (0.025)	0.14 (0.02)		188	<0.001	0.83
Lapillus *vs*. age	61	1.4 (0.06)	0.594 (0.024)	0.10 (0.02)	−0.06 (0.02)	218	<0.001	0.92
Aster. *vs*. age	73	2.3 (0.06)	0.448 (0.022)	0.13 (0.02)	NS	162	<0.001	0.88
Aster. *vs*. age p.h.	73	2.3 (0.06)	0.448 (0.022)	0.13 (0.02)		247	<0.001	0.88
Sagittal *vs*. BM	80	4.1 (0.05)	0.707 (0.077)	NS	NS	40	<0.001	0.61
Sagittal *vs*. BM p.h.	80	4.0 (0.05)	0.736 (0.068)			116	<0.001	0.60
Lapillus *vs*. BM	61	2.4 (0.06)	0.978 (0.100)	NS	NS	52	<0.001	0.64
Lap. *vs*. BM p.h.	61	2.4 (0.06)	0.908 (0.092)			98	<0.001	0.62
Aster. *vs*. BM	73	3.1 (0.05)	0.739 (0.077)	NS	NS	42	<0.001	0.64
Aster. *vs*. BM p.h.	73	3.0 (0.05)	0.741 (0.068)			118	<0.001	0.62

*Note*: Aster, asteriscus; *I*, *y* intercept; NS, not significant; *m*, slope; p.h., post‐hoc; SexF, shift in *y* intercept of females compared to males; Sex*AgeF, change in *m* of females relative to males. Number in parenthesis beside estimate is the standard error.

**FIGURE 7:**
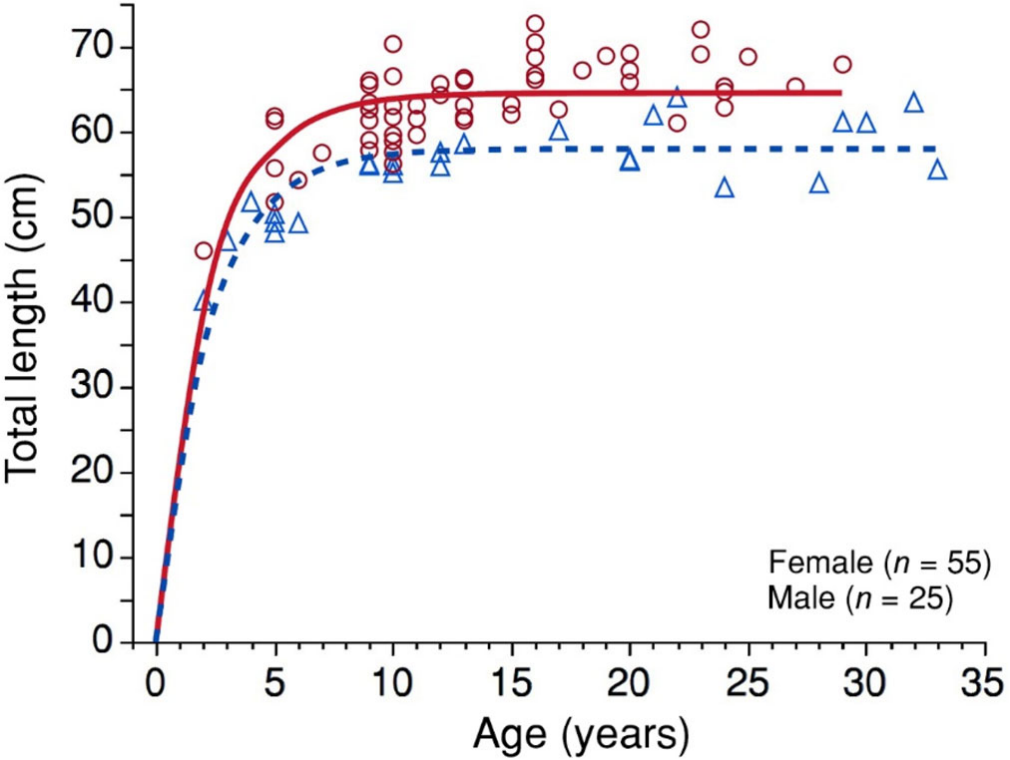
Total length versus age for female (circles) and male (triangles) bowfin *Amia calva* in this study for which a length and age was determined (*n =* 80; one of our 81 specimens was not measured for TL). Shown is the highest‐ranked von Bertalanffy growth model (*F*_3,76_ = 73.6, d.f. = 4, *P* < 0.001, *R* ^2^ = 0.66) for females (solid red line) and males (dotted blue line) in which parameters for asymptotic length (*L*_*∞*_) differed by sex [females: *L*_*∞*_ = 64.5, 95% CI (63.4, 65.7); males: *L*_*∞*_ = 58.0 (56.1, 59.9)] but instantaneous growth rate [*k* = 0.454 (0.386, 0.549)] did not; age at 0 length was fixed (*t*_0_ = 0)

Bowfin exhibit sexual dimorphism in asymptotic body length, but not in their rate of somatic growth. In the highest‐ranked von Bertalanffy growth model (Figure 7 and Table 3), the estimated *L*_*∞*_ was 64.5 cm for females compared to 58.0 cm for males, but there was no difference in the instantaneous rate of increase (*k* = 0.454). According to this model, Bowfin reach ~98% of asymptotic length by an estimated age of 9 years and may live decades at asymptotic size (Figure 7). We found that the early‐life growth rate (*ω*) was 29.3 cm/year for females *vs*. 26.3 cm/year for males.

**TABLE 3:** Model selection statistics for all von Bertalanffy growth functions in the TL *vs.* Age model suite, (*n* = 80)

Model	SSE	*k*	AICc	ΔAICc	*F*	*P*	*R* ^2^
*k* NSS, *t*_*0*_ = 0	1126	3	220.1	0.0	74	<0.001	0.656
Global with *t*_*0*_ = 0	1116	4	221.7	1.6	49	<0.001	0.659
*L*_∞_ NSS, *t*_0_ = 0	1646	3	250.5	30.4	38	<0.001	0.498
*L*_∞_, *k* NSS, *t*_0_ = 0	1796	2	255.2	35.1	64	<0.001	0.452

*Note*: Global has sex‐specific *L*_∞_ and *k*. NSS, not sex‐specific.

**FIGURE 8:**
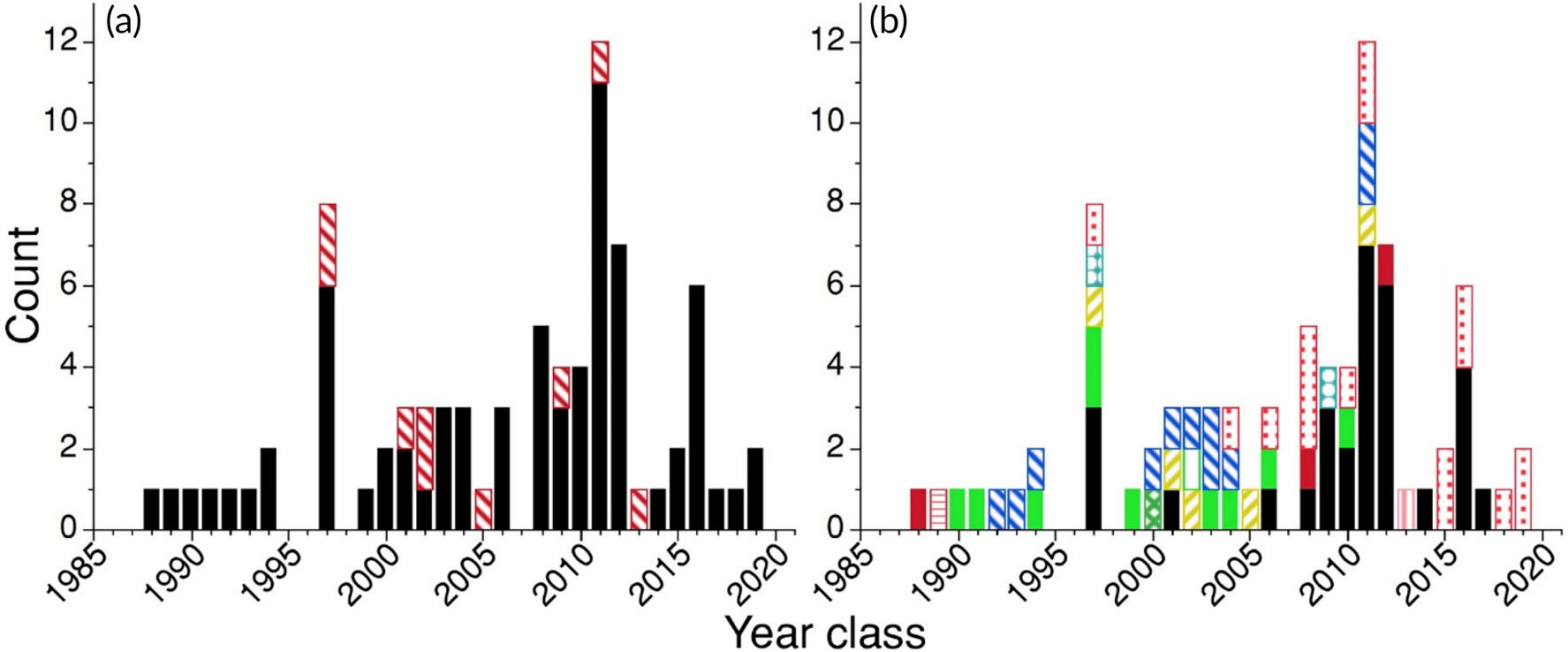
Year class distribution of otolith‐aged Bowfin *Amia calva* (*n* = 81) collected by bowfishers from 2017 to 2021 in Minnesota from 11 sites across a narrow latitudinal gradient (<50 km) by drainage (a) and waterbody (b). 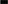 Mississippi. 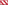 Red. 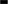 Big Sandy. 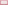 Cedar. 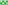 Clark. 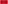 Lower Island. 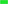 Lower Cullen. 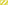 Little Pelican. 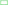 North Lida. 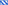 Riverton. 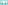 Rush. 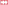 Tamarack. 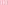 West Lost

**FIGURE 9:**
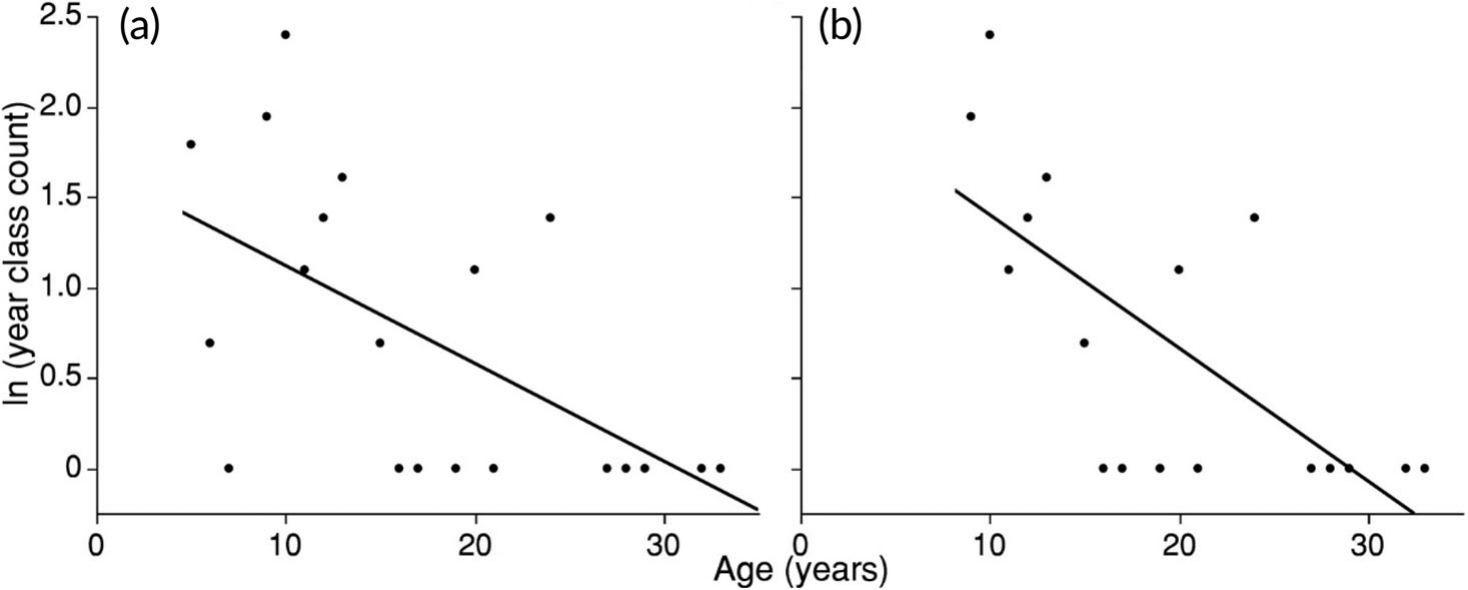
Catch curves of natural log transformed year class count versus age of bowfin collected in 2021 assuming (a) bowfin recruit to the fishery by an age of 5 years (*F*_1,18_ = 9.3, d.f. = 1, *P* = 0.007, *R* ^2^ = 0.34 based on a sample of *n* = 57 fish) with estimated annual mortality rate of *A* = 5.3%, and (B) bowfin recruit to the fishery by an age of 9 years (*F*_1,15_ = 14.4, d.f. = 1, *P* = 0.002, *R* ^2^ = 0.49) suggesting a higher annual mortality rate of *A* = 7.1%

Catch curve analysis indicated variable bowfin recruitment, with annual mortality rate of 5%–7% for recruits. Estimated year classes in this study ranged from 1988 to 2019 for fish collected from 2017 to 2021 (with 74% collected in 2021) (Figure 8). The 1997 and 2011 year classes comprised 25% of the sample, and were the only year classes represented at four or more sites (Figure 8b). Although 44% of the bowfin collected by bowfishers from Tamarack Lake were estimated to be 2–6 years old, 89% of the bowfin collected by bowfishing from other sites were estimated to be 9 years or older (Figure 8b). The RCD for bowfin was 0.34 if individuals recruit by age 5 and 0.49 if individuals recruit by age 9 (Figure 9), values which Isermann *et al*. (2002) classify as moderately variable recruitment. We estimated an annual mortality of 5.3% if individuals recruit to the bowfishery by an estimated age of 5 years and 7.1% if individuals recruit to the bowfishery by an estimated age of 9 years (Figure 9).

## 5 DISCUSSION

To our knowledge, this is the first study in which otoliths have been successfully used to estimate the age of bowfin. Evidence from presumed annuli in thin‐sectioned otoliths indicates that previously reported longevities for bowfin from wild populations are perhaps underestimates, but this remains to be conclusively determined. We found evidence that suggests bowfin may live 33 years, which is 15 years older than the maximum age previously estimated for wild populations (Mundahl *et al*., 1998). Interestingly, several captive bowfin for which actual ages are known (20–30 years; Breder, 1936; Flower, 1925, 1935) are comparable in lifespan to what we estimate in this study, which supports the credibility of our otolith‐derived age estimates. In this study we estimate that more than 25% of the bowfin collected were at least 20 years old, with some individuals exceeding 30 years of age. We conclude that evidence indicates bowfin may live multiple decades in the wild, contrary to all reports from other wild populations of this species, all of which used non‐otolith structures to estimate ages.

Previous studies on bowfin population demographics are characterized by younger age distributions, with age estimations based on analysis of structures other than otoliths. Of a combined total of 1116 bowfin (TL ranging from 22.1 to 80.7 cm) aged using non‐otolith structures (Cartier & Magnin, 1967; Cooper & Schafer, 1954; Daniels, 1993; Davis, 2006; Hausmann, 1998; Holland, 1964; Koch *et al*., 2009a, 2009b; Mundahl *et al*., 1998; Porter *et al*., 2014; Sanderson‐Kilchenstein, 2015; Schiavone Jr., 1982), at least 1068 (≥96%) were estimated to be 13 years or younger, and none was more than 18 years old (Table 1). Age estimates derived from hard parts other than otoliths have been shown repeatedly to underestimate otolith‐determined ages in a number of species (Beamish & McFarlane, 1983, 1987; Campana, 2001; Casselman, 1990; Lackmann *et al*., 2021a; Radford *et al*., 2021; Sylvester & Berry Jr., 2006), including holosteans (Buckmeier *et al*., 2018). Our findings suggest this may be the case for bowfin as well, but a future study that tests the validity of age estimates in bowfin is necessary to prove if this is has likely been the case.

Prior to our findings, attempts to estimate age from otoliths in bowfin have been unsuccessful. Several previous attempts (Cartier & Magnin, 1967; Davis, 2006; Koch *et al*., 2009a) abandoned use of otoliths due to unspecified difficulties with these structures. Cartier and Magnin (1967) do not specify which otolith type (sagitta, asteriscus or lapillus) was used, but regardless did not proceed with whole otoliths and instead used bowfin scales. We assume Cartier and Magnin (1967) used the sagittae because these are the largest otoliths in bowfin. Davis (2006) extracted sagittae and viewed them both whole and sectioned under a dissecting microscope, but was unable to estimate age from either approach. Koch *et al*. (2009a) reference the extraction of all otolith types, viewing thin sections under a dissecting microscope, but were also unable to use bowfin otoliths to estimate age. Neither Davis (2006) nor Koch *et al*. (2009a) report how many fish were examined, and Koch *et al*. (2009a) do not report which otolith type(s) they used before abandoning efforts to estimate bowfin age using the otolith.

Contrary to these earlier studies, we used all otolith types for age estimation of bowfin. We conclude that lapillus otoliths are the most useful structure because 100% of thin sections were readable, followed by asterisci in which 90% were readable and then sagittae in which 60% were readable. This despite the lapillus being the smallest otolith in bowfin. Typically, the largest otolith type is used in age analysis because it is the easiest to extract and process. However, we found the lowest utility for bowfin sagittae according to percentage of otolith sections that were readable.

We evaluated otolith allometry, which helped inform why lapillus and asteriscus otoliths from bowfin are more useful in age estimation compared to bowfin sagittal otoliths. Allometric patterns of otolith growth in bowfin are consistent with our findings about the relative reliability of different otolith types for age estimation in this species. As a bowfin grows older and larger, mass of the lapilli increases at a higher rate than mass of either the asterisci or sagittae. Thus, proportionately more otolith growth happens in the bowfin lapillus as the fish ages and grows, which results in relatively more space to distinguish presumed annuli along the primary growth axis of a thin section. Based on these findings, we recommend analysing whole otolith mass as a function of body mass (and estimated age, if known) among otolith types to guide selection of which otolith type to use for age estimation rather than using absolute otolith size alone. Allometric analysis may aid otolith type assessments in other species with similar otolith characteristics. In addition, high opacity in bowfin sagittae was more likely to obscure presumed annuli compared to the other otolith types, presumably due to higher concentrations of the aragonitic polymorph of calcium carbonate noted in the sagittal otoliths of other species (Campana, 1999, 2005). On the other hand, the asteriscus is known to be primarily composed of vateritic calcium carbonate, which is more translucent (Campana, 1999), and this may explain why presumed annuli were more discernible in bowfin asterisci, compared to their generally more opaque sagittae.

We found that bowfin grow to 40–50 cm total length in their estimated first 2–3 years of life, and reach asymptotic lengths by an estimated age of 8–10 years. We presume that non‐otolith structures such as scales, gular plates and fin rays plateau in growth similar to the linear axis of the skeleton and discernible annuli diminish around this same age threshold. Interestingly, the maximum age of other bowfin studies was typically 10 years (Table 1) using non‐otolith structures. Previous work on bowfin documented higher maximum ages for female bowfin than for males (Davis, 2006; Koch *et al*., 2009a; Porter *et al*., 2014; Sanderson‐Kilchenstein, 2015). In this study we found that the three oldest bowfin (30–33 years) were males. Bowfin size is sexually size‐dimorphic (with males smaller than females), consistent with previous findings (Cartier & Magnin, 1967; Cooper & Schafer, 1954; Daniels, 1993; Davis, 2006; Hausmann, 1998; Holland, 1964; Koch *et al*., 2009a, 2009b; Mundahl *et al*., 1998; Porter *et al*., 2014; Sanderson‐Kilchenstein, 2015; Schiavone Jr., 1982). However, bowfin growth rates (*k* = 0.45) from the von Bertalanffy models reported here correspond to smaller total length at age, and therefore a slower growth rate, than reported for other populations (Cartier & Magnin, 1967; Cooper & Schafer, 1954; Daniels, 1993; Davis, 2006; Hausmann, 1998; Holland, 1964; Koch *et al*., 2009a, 2009b; Porter *et al*., 2014; Sanderson‐Kilchenstein, 2015; Schiavone Jr., 1982). Age estimation using non‐otolith structures may be more likely to underestimate ages of male bowfin, in light of otolith allometry and males' smaller body size. This should be tested. Given that bowfin may be entering the bowfishing fishery as early as 4–5 years of age, new information on gonad size at age using otolith‐estimated ages is needed to estimate rates of sexual maturation in bowfin relative to their vulnerability to harvest.

The year class distribution and recruitment analysis from otolith‐aged bowfin suggests recruitment of this species is more complex and variable than patterns reported in prior studies, where ages were derived from hard parts other than otoliths (*e.g*., Koch *et al*., 2009b). Again however, these recruitment results are contingent on the assumption of unbiased harvest beyond a certain threshold (Isermann *et al*., 2002). Since such assumptions have yet to be tested for bowfishing, the recruitment results we document should be interpreted with caution. More otolith‐based age data are needed to understand bowfin recruitment patterns, both within and among sites, and across the species' range. We note that this study's sample was from a pooled bowfishing sample, which presents a non‐negligible risk of being a size‐selective sample that is limited in its recruitment inferences (*e.g*., the estimates are conditional on the assumption that the sample can be generalized as a single population). Thus, the recruitment results that we document require caution in interpretation. Nonetheless, bowfishing is a new and growing sportfishery in need of management (Bennett *et al*., 2015; Lackmann *et al*., 2019, 2022; Lackmann *et al*., 2021b; Quinn, 2010; Scarnecchia *et al*., 2021; Scarnecchia & Schooley, 2020, 2022; York *et al*., 2022), so the bowfishing‐specific results that we document are especially relevant. Bowfin are increasingly being targeted by bowfisheries nationwide, with most targeted fish being discarded (Scarnecchia & Schooley, 2020), while also a growing target of a caviar industry (Sinopoli & Stewart, 2021). We recommend that efforts be made to obtain otolith data from discarded bowfin. Since otoliths are widely documented as the most accurate structure for estimating age across fishes (Campana, 2001), and bowfin otoliths were not thoroughly analysed before, the data that we present could possibly be the most credible age‐demographic information on this species to date. Nonetheless, future study is needed to definitively document if this is the case (*e.g*., age validation; Beamish & McFarlane, 1983).

Further study of bowfin population demographics is warranted throughout their range, especially in the context of modern harvest pressures. The ability to sustainably manage fish stocks requires an accurate understanding of the life history and the exploitation rates of the species involved, neither of which is well understood for bowfin. Crucial next steps for reassessing the ecology of bowfin include (1) validating the accuracy of otolith‐estimated bowfin ages, (2) investigating bowfin age structure using otoliths from other populations, (3) obtaining information on otolith‐estimated population age structure and age at maturity, and (4) quantifying bowfishing harvest rates while capitalizing on data available from discarded fish (*e.g*., study bowfin shot at tournaments). We demonstrate a process to estimate bowfin ages from their otoliths, particularly using thin‐sectioned otoliths, which sets the stage for future age validation work.

Bowfin management is overdue (Scarnecchia, 1992), particularly as this species has become a targeted sportfish with the rise of modern bowfishing (Scarnecchia *et al*., 2021; Scarnecchia & Schooley, 2020). The approach we outline here, in which bowfishing discards are used to evaluate population structure using age estimates from otoliths, and using otolith allometry to inform otolith type assessments, also provides a blueprint for evaluations in other species now vulnerable to this rapidly growing sport fishery (*e.g*., Bennett *et al*., 2015; Lackmann *et al*., 2019, 2022; Quinn, 2010). Species like bowfin generally have no limits on their take and continue to be derogatorily classified as “rough fish” (Rypel *et al*., 2021; Sinopoli & Stewart, 2021), despite being important to the native environment of North America (Scarnecchia, 1992; Burr & Bennett, 2014). Unlimited take of wild freshwater fish with no active management amidst increasingly popular and efficient forms of exploitation warrants attention. We are just beginning to understand the complexities of these native species and the roles they play in dynamic ecosystems.

## ETHICS STATEMENT

We have treated all animals in accordance with North Dakota State University and University of Minnesota Duluth Institutional Animal Care and Use Committee (IACUC) guidelines on animal care as specified in each protocol (IACUC protocols A17007 and 2007‐38272A). The collection of specimens was conducted in accordance with all applicable laws, guidelines and regulations. Specimen collection permits for this study were granted by the Minnesota Department of Natural Resources (fisheries research licence numbers: 23775, 28375, 29788, 32489).
